# Season of Birth Impacts the Neonatal Nasopharyngeal Microbiota

**DOI:** 10.3390/children7050045

**Published:** 2020-05-11

**Authors:** Ann-Marie Malby Schoos, Marie Kragh, Peter Ahrens, Katrin Gaardbo Kuhn, Morten Arendt Rasmussen, Bo Lund Chawes, Jørgen Skov Jensen, Susanne Brix, Hans Bisgaard, Jakob Stokholm

**Affiliations:** 1 COPSAC, Copenhagen Prospective Studies on Asthma in Childhood, Herlev and Gentofte Hospital, University of Copenhagen, Ledreborg Alle 34, 2820 Gentofte, Denmark; ann-marie.schoos@dbac.dk (A.-M.M.S.); morten.arendt@dbac.dk (M.A.R.); chawes@copsac.com (B.L.C.); sbp@bio.dtu.dk (S.B.); stokholm@copsac.com (J.S.); 2 Department of Biotechnology and Biomedicine, Technical University of Denmark, 2800 Lyngby, Denmark; ymk@novonordisk.com; 3 Microbiology and Infection Control, Sexually Transmitted Infections, Statens Serum Institut, 2300 Copenhagen, Denmark; pae@ssi.dk (P.A.); kuh@ssi.dk (K.G.K.); jsj@ssi.dk (J.S.J.); 4 Faculty of Science, University of Copenhagen, 1958 Frederiksberg, Denmark

**Keywords:** season of birth, nasopharynx, microbiota, bacterial richness, bacteria, summer, children, terminal restriction fragment length polymorphism

## Abstract

**Objective**: Pathogenic airway bacteria colonizing the neonatal airway increase the risk of childhood asthma, but little is known about the determinants of the establishment and dynamics of the airway microbiota in early life. We studied associations between perinatal risk factors and bacterial richness of the commensal milieu in the neonatal respiratory tract. **Methods**: Three hundred and twenty-eight children from the Copenhagen Prospective Studies on Asthma in the Childhood2000 (COPSAC2000) at-risk birth cohort were included in this study. The bacterial richness in each of the nasopharynxes of the 1-month old, asymptomatic neonates was analyzed by use of a culture-independent technique (T-RFLP). Information on perinatal risk factors included predisposition to asthma, allergy and eczema; social status of family; maternal exposures during pregnancy; mode of delivery; and postnatal exposures. The risk factor analysis was done by conventional statistics and partial least square discriminant analysis (PLSDA). **Results**: The nasopharyngeal bacterial community at 1-month displayed an average of 35 (IQR: 14–55, range 1–161) phylogenetically different bacteria groups. Season of birth was associated with nasopharyngeal bacterial richness at 1-month of age with a higher bacterial richness (p = 0.003) and more abundant specific bacterial profiles representing Gram-negative alpha-proteobacteria and Gram-positive Bacilli in the nasopharynx of summer-born children. **Conclusion:** Early postnatal bacterial colonization of the upper airways is significantly affected by birth season, emphasizing a future focus on the seasonality aspect in modelling the impact of early dynamic changes in airway bacterial communities in relation to later disease development.

## 1. Introduction

Asymptomatic colonization in neonates with the common respiratory tract pathogens *Haemophilus influenzae, Moraxella catarrhalis* and *Streptococcus pneumoniae* has been shown to confer a higher risk of subsequent lung diseases, including childhood asthma [1,2,3]. The respiratory tract pathogenic bacteria operate in a gene-environment matrix with the airway mucosa and commensal bacteria, possibly impacting respiratory health by inducing early life topical immune deregulation [4,5]. Still, little is known about the establishment and dynamics of the commensal microbiota in the neonatal respiratory tract [6] and the influences of genetic and perinatal environmental factors. 

Complex bacterial communities, such as those within the respiratory tract, have been studied by a number of different methods, including cultivation, which may be selective for certain species, but more recently, profiling of airway bacterial communities by culture-independent DNA based methods such as terminal restriction fragment length polymorphism (T-RFLP) [7,8] has been applied. We aimed to determine the nasopharyngeal bacterial richness by the T-RFLP technique in 1-month old children of the Copenhagen Prospective Studies of Asthma in Childhood_2000_ (COPSAC_2000_) cohort. Fecal bacterial richness has previously been related to both childhood allergies [9], and metabolic disorders in adults [10], thereby illustrating that bacterial richness may be of importance for several pathophysiological processes. In this study we focused on factors that could potentially influence the richness of the nasopharyngeal microbiota during establishment in early life, including perinatal risk factors such as predisposition to asthma and allergies and environmental risk factors relevant for the establishment of a commensal milieu in the respiratory tract. Season of birth has previously been related to lifetime disease risk for many different conditions [11]. Among these, associations have been observed for asthma, allergic sensitization [12] and food allergies in particular [13], with potential mediation through differences in commensal microbiota in the respiratory tract influencing the early immune system’s development [5].

## 2. Methods

### 2.1. Study Population 

COPSAC_2000_ is an ongoing prospective longitudinal birth cohort study of 411 children born to mothers with histories of asthma [14] recruited from Zealand, Denmark in the years 1998–2001. The children attended the COPSAC Clinical Research Unit at 1-month of age and were followed prospectively with clinical visits every 6 months until the age of 7. Exclusion criteria were a severe congenital anomaly, a gestational age younger than 36 weeks, a need for mechanical ventilation and a lower respiratory tract infection. 

### 2.2. Ethics Statement

The study was conducted in accordance with the Declaration of Helsinki and approved by the Copenhagen Ethics Committee (KF01-289/96) and the Danish Data Protection Agency (2015-41-3696). Data collection was in accordance with Good Clinical Practice guidelines. Written informed consent was obtained from both parents before enrolment of the children. 

### 2.3. Collection of Nasopharyngeal Samples at 1-Month of Age

Nasopharyngeal specimens were collected by the COPSAC physicians from September 1999 to December 2001 from asymptomatic neonates during the scheduled 1-month visit. The specimens were obtained from the posterior nasopharynx by using an ENT cotton-tipped aluminum swab (Medical Wire and Equipment, Corsham, UK) for 15 s and rotating it two times before it was retracted and placed in 1.8 mL of SP4 mycoplasma transport medium [15,16]. The samples were kept at +4 °C until they were delivered to the laboratory the next day, where they were processed for mycoplasma culture as part of another protocol. The remaining sample material was immediately frozen at −80 °C and was not thawed before it was used for DNA extraction.

### 2.4. DNA Extraction from Nasopharyngeal Specimens for T-RFLP 

Bacteria from nasopharyngeal swabs were harvested by centrifugation of 500 µL each of specimens at 30,000 *g* for 15 min. Each pellet was resuspended in 200 µL TE buffer (30 mM Tris-HCl, 1 mM EDTA, pH 8.0) with 15 mg/mL lysozyme (Sigma-Aldrich, Seelze, Germany) and 20 µg/mL protease (Qiagen, Hilden, Germany) and mixed with 200 µL AL lysis buffer (Qiagen) and zirconium beads (BioSpec Products, Bartlesville, OK) prior to disruption and homogenization in a MagNa Lyser Instrument (Roche Applied Science, Germany) for 70 s at setting 7000. This was followed by 10 min incubation at 56 °C. The samples were centrifuged at 30,000 *g* for 5 min, and the DNA was purified using the Qiagen DNeasy blood and tissue kit according to the manufacturer’s instructions. In brief, the DNA extract was mixed with ethanol, applied to the spin column and centrifuged at 8000 *g* for 1 min in order to bind DNA in the lysate to the column. Then, the column was washed with Buffer AW1 (8000 *g*, 1 min) followed by another wash with Buffer AW2 (14,000 *g*, 3 min). Finally, DNA was eluted with 200 µL (2 × 100 µL) Buffer AE (8000 *g*, 1 min). The eluted DNA was stored at −80 °C.

### 2.5. T-RFLP on Nasopharyngeal Specimens from Infants

Our T-RFLP protocol was based on amplification of the 16S rRNA gene, which has been widely used for resolving bacteria and their diversity across biological systems and molecular techniques [17]. We applied one fluorescently labelled primer and the restriction enzyme *HhaI* being aware of issues with underestimation of the microbial diversity [18,19]. For binning of sized terminal restriction fragments (TRFs), we used T-REX [20], which has recently been demonstrated to be usable in T-RFLP analysis on microbial upper airway specimens [7,8]. 

From the extracted DNA, the V1–V3 regions of the 16S rRNA gene were PCR amplified using the fluorescein (FAM)-labelled primer 07F-5’-AAGAGTTTGATCATGGCTCA-3’ and the unlabeled 535R-5’-GTATTACCGCGGCTGCTGG-3’ (TAG Copenhagen, Copenhagen, Denmark). The PCR product was generated using a 25 µL PCR mixture containing 20 mM Tris/HCl (pH 8.4), 50 mM KCl, 125 µM dNTP mix, 1.5 mM MgCl_2_, 5 pmol of each primer (5-FAM™ 07F and 535R) and 0.5 U Platinum^®^ Taq polymerase (Life Technologies), and 1 µL of extracted DNA. Amplification was performed in an ABI 2720 Thermal Cycler (Life Technologies) and consisted of a denaturation at 94 °C for 4 min followed by cycles of denaturation at 94 °C for 30 s, annealing at 50 °C for 30 s and extension at 72 °C for 1 min. Amplicons were visualized by electrophoresis on 1.5% agarose gels, stained with ethidium bromide and examined by UV trans-illumination. In order to minimize amplification bias, all samples were initially amplified for 28 cycles; for samples with weak or no visible amplicons after 28 cycles, PCR was repeated with 32 and 35 cycles respectively. The PCR product was precipitated with 96% ethanol, and then washed twice in 70% ethanol. Remaining ethanol was evaporated, and the pellet resuspended in 10 µL TE-buffer for storage at 4 °C. The PCR setup work area and the amplified DNA work area were physically separated to avoid PCR product carryover. 

Prior to treatment with restriction enzyme, excess primers and nucleotides were removed by incubation of 2 µL sample with 1 µL 10× NEBuffer 4-restriction buffer (New England BioLabs Inc., Hertfordshire, UK), 6.5 µL water and 0.5 µL ExoSAP (USB Corporation, Cleveland, OH) at 37 °C for 15 min, followed by inactivation of the enzyme at 72 °C for 15 min. The mixture was supplemented with 2 µL 1× NEBuffer 4 containing one unit of restriction enzyme *HhaI* (GCG^C), (New England BioLabs Inc., Ipswich, MA), and left at 37 °C for 2 h for enzymatic cleavage. The digested DNA was precipitated by ethanol, dried and resuspended in 10 µL HiDi formamide (ABI) containing 0.01 µL MegaBace-900ROX™ marker (Amersham). The size standard contained 37 bands at 25 bp periodicity with feature bands at 60, 310 and 610 bp.

Samples were denatured for 1 min at 94 °C and analyzed on an Applied Biosystems^®^ 3130*xl* Genetic Analyzer with Data Collection Software v3.0 (Life Technologies). The resulting T-RFLP electropherograms (fsa files) were uploaded to the Peak Scanner Software v1.0 for sizing of terminal restriction fragments (TRFs). A baseline of 35 FU was applied to eliminate background noise, and peaks were detected and size-matched by use of a size-calling curve generated by the size-calling method “Local Southern algorithm.” The resulting sizing table was exported and uploaded to the T-RFLP analysis *EX*pedited software (T-REX) [20] in order to align TRFs. In T-REX, clustering threshold of 1.0 was used in the alignment process. TRFs present in <1% of the nasopharyngeal specimens were omitted from further analysis. The complex nasopharyngeal specimens of varying DNA content limited a robust evaluation of peak abundances. The richness of bacterial groups within nasopharyngeal specimens at 1-month of age was therefore the sole output parameter from T-RFLP. 

### 2.6. Identification of Bacterial Groups with Given TRFs

To identify which bacteria groups the observed TRFs could be part of, we used the software MiCA (http://mica.ibest.uidaho.edu/T-RFLP.php) [21], while assuming that experimental results would differ by 5 bp [22]. 

### 2.7. Perinatal Risk Factors

The risk factor analysis included baseline information on atopic predisposition based on doctor-diagnosed parental asthma, rhinitis and eczema. Baseline information on perinatal risk factors, including family income, mother’s education (college/elementary/university) and occupation (unemployed/non-professional/professional/student), was investigated together with a range of exposures during pregnancy comprising use of antibiotics, use of paracetamol, smoking and alcohol consumption in the 3rd trimester. The impact of previous deliveries on bacterial richness was also assessed. Risk factors at birth included gender, mother’s age at birth, gestational age, mode of delivery (natural birth/Caesarean section), Apgar score (≥9 after 5 min) and season of birth (December, January, February = winter; March, April, May = spring; June, July, August = summer; September, October, November = autumn). Risk factors after birth included exposures to cats/dogs in the home and breastfeeding. Information was recorded by personal interviews by the trained staff at scheduled visits at the COPSAC Clinical Research Unit. 

### 2.8. Statistical Analysis 

Pearson’s chi-squared test and unpaired *t*-test were employed for simple baseline statistics. For each child, the final T-RFLP data were reported as a profile consisting of binomial data, indicating the presence (1) or absence (0) of a certain TRF. The numbers of different TRFs defined bacterial richness. In order to reach normal distribution, the richness value was square-root transformed. Associations between perinatal risk factors and TRF richness in the nasopharynxes of asymptomatic neonates were analyzed by ANOVA (factor variables) and linear regression (continuous variables). Statistical significance was defined by *p* < 0.05. Data processing was performed with R v2.15.3 (http://www.R-project.org, R Development Core Team, Vienna, Austria). 

Partial least square discriminant analysis (PLSDA) was employed to attain latent variables that could discriminate the TRF pattern of infants by risk factor. PLSDA can analyze data with strongly correlated, noisy and numerous X-variables (here TRFs), and simultaneously model the response Y-variable [23]. In order to not over-fit the PLSDA model, thereby including un-systematic variation, cross-validation of 10 splits with random segmentation for 20 iterations was applied. Random permutation testing with 10,000 permutations was conducted to calculate the probability of model insignificance versus permuted samples. PLSDA and permutation testing were done in MATLAB R2010b v7.11.0.584 (Mathworks, Natick, MA) with PLS_Toolbox v7.5.2 (Eigenvector Research, Wenatchee, WA).

## 3. Results

### 3.1. Characteristics of Study Population

Collection of nasopharyngeal specimens was initiated after enrolment of 47 of the 411 children in the cohort, resulting in 364 collected samples. Thirty-six specimens were lost in delivery or for technical reasons, providing nasopharyngeal specimens from 328 (80%) infants born over a time-period of two years. The median age of the infants at specimen collection was 42 (IQR: 34–54) days. Baseline comparisons between the children with (*n* = 328) and without available specimens (*n* = 83) are reported in Table S1. A lower percentage of children in the study cohort compared to the remaining children had mothers using paracetamol (12% vs. 24%, *p* = 0.01) and alcohol (16% vs. 27%, *p* = 0.03) in the 3rd trimester. Moreover, lower percentages of the children in the study cohort were exposed to dogs at birth (10% vs. 18%, *p* = 0.04) and were exclusively breastfed the first 30 days postnatally (60% vs. 72%, *p* = 0.04) than not.

### 3.2. Nasopharyngeal Bacterial Richness in Asymptomatic Neonates

**Figure 1:**
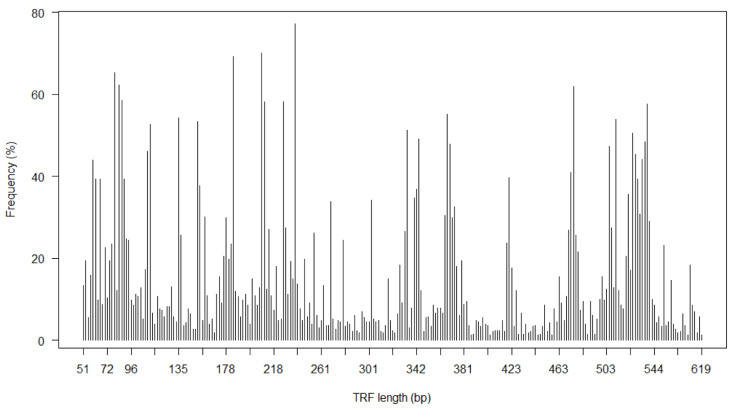
Nasopharyngeal bacterial richness in 1-month old children. Frequency of terminal restriction fragments (TRFs) obtained by *HhaI* cleavage of 16S rRNA gene isolated from nasopharyngeal swab specimens of 328 asymptomatic 1-month old neonates. The TRFs are displayed after binning and alignment of TRFs from individual samples.

For each child, a community profile composed of phylogenetically distinct bacterial groups (TRFs) was generated with the number of different TRFs as a measure of bacterial richness. We focused on TRFs prevalent in >1% of the 328 neonates, resulting in a total number of 261 different TRFs (15 TRFs occurred in <1% of the specimens) (Figure 1). The TRF median richness was 35 (interquartile range 14–55 TRF, range 1–161). 

### 3.3. Predictors of Neonatal Nasopharyngeal Bacterial Richness 

TRF richness of the neonatal nasopharynx was compared to perinatal factors, including predisposition to asthma and allergy, social status of family, pregnancy history, birth-related factors and environmental exposures (Table 1). 

Family income was significantly associated with bacterial richness (*p* = 0.031), with lower bacterial richness being present for families with low incomes. A mother’s education and occupation were not associated with bacterial richness. 

Amongst parental disease risk factors, paternal dermatitis was marginally associated with nasopharyngeal bacterial richness (*p* = 0.044), with infants of fathers with dermatitis displaying a higher richness. Paternal rhinitis and asthma showed similar trends but were not significant. None of the pregnancy risk factors were associated with nasopharyngeal TRF richness in 1-month old infants. 

**Figure 2:**
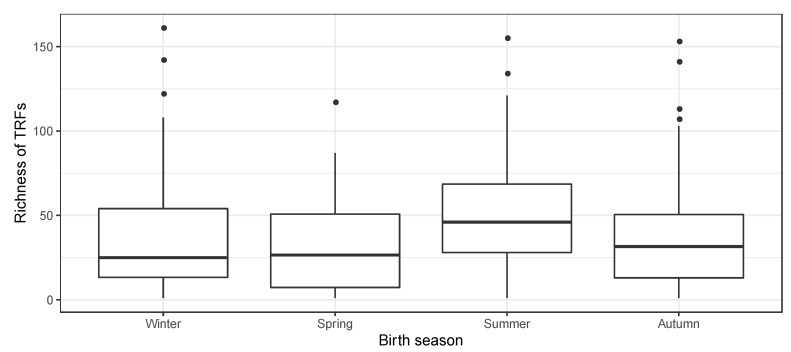
Season of birth and bacterial richness. Number of terminal restriction fragments (TRFs) observed per airway sample in 328 asymptomatic 1-month old neonates.

Season of birth was found to significantly influence nasopharyngeal TRF richness (ANOVA *p* = 0.003, Kruskal–Wallis *p* = 0.003). The TRF richness was higher in infants born in the summer compared to infants born in the autumn (*p* = 0.032), winter (*p* = 0.058) and spring (*p* = 0.003) (Figure 2). Multiple linear regression analysis including dermatitis in the father and family income did not affect the birth season’s association with nasopharyngeal bacterial richness (*p* = 0.008). 

The postnatal factors, including at-birth exposures to older siblings, cats and dogs did not associate with bacterial nasopharyngeal richness. Moreover, TRF richness was not affected by the child’s age during specimen collection. 

After false discovery rate (FDR) correction at a 5% level, only season of birth was still found to be significantly associated with TRF richness (*p* = 0.035), whereas family income and paternal dermatitis were found to be less likely to influence nasopharyngeal bacterial richness (*p* = 0.25 and *p* = 0.24, respectively). We therefore focused the following post-validation tests on birth season relations to nasopharyngeal bacterial richness.

### 3.4. Season of Birth and Nasopharyngeal Bacterial Richness in Neonates

To test the robustness of the birth season association to nasopharyngeal bacterial richness, we examined whether the seasonal variation persisted after removal of 31 TRFs with low abundance (present in < 10% of the samples). Again, we found season of birth associated with nasal bacterial richness (*p* = 0.005) with higher richness in infants born in summer compared to infants born in autumn (*p* = 0.007), winter (*p* = 0.010) and spring (*p* = 0.001). 

Children within the study cohort were born over two consecutive years, allowing for testing of seasonal consistency in specimen collection by dividing data into two subsets representing two different consecutive time-periods of one year. Again, we confirmed a higher bacterial richness in summer-born children compared to autumn, winter and spring-born children across the time-periods (Table S2). The bacterial richness IQR was equally distributed over seasons, justifying the fact that higher individual richness in a few samples was not responsible for the overall bacterial richness assigned to season (Figure S1). 

### 3.5. Discriminatory Nasopharyngeal Bacterial Richness in Relation to Season of Birth 

**Figure 3:**
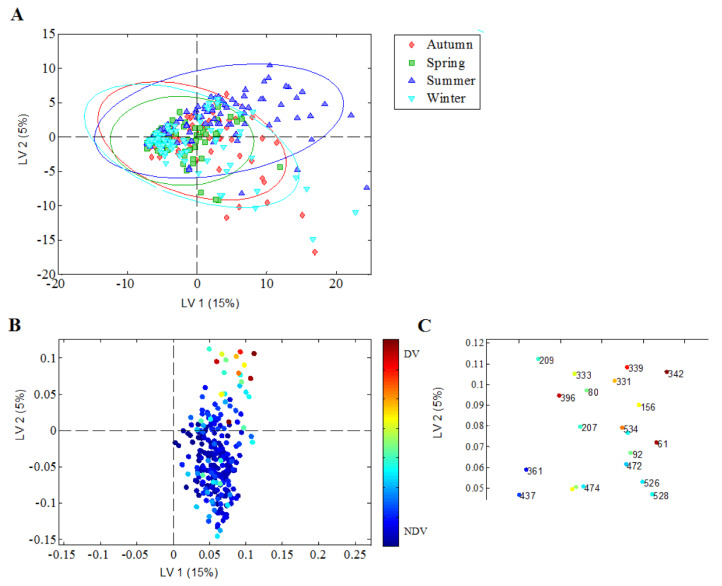
Season of birth-related bacterial richness with a focus on TRFs represented in children born in the summer season. The TRFs were obtained by *HhaI* cleavage of 16S rRNA gene isolated from nasopharyngeal swab specimens. TRFs profiles were modelled by partial least square regression with season of birth as the discriminatory variable. (**A**) Scores plot. The resulting PLSDA model with two discriminatory latent variables (LVs) explained 20% of the total variation in TRF pattern had a cross-validated sensitivity and specificity of 0.68 (*p*_permutation,cross-validated_ < 0.001), and was able to discriminate summer-born children from autumn, winter and spring-born children. Each child is represented by one symbol. Ellipses represent 95% of the class distribution. (**B**) Loadings plot. Each TRF is represented by a dot. TRFs with discriminatory power (DV) and TRFs of non-discriminatory power (NDV) are marked by differing color intensity. (**C**) Zoomed image of loadings plot for TRFs dominant in summer-born children. Identification of TRFs, shown as length in bp, with discriminating power to separate bacterial profiles of summer-born children from autumn, winter and spring-born children.

In order to identify whether certain TRFs were more prevalent in children born in the summer season compared to children born in autumn, winter and spring, we applied the data-driven method of PLSDA. The resulting PLSDA model with two discriminatory latent variables (LVs) explained 20% of the total variation in the TRF pattern. The model was able to discriminate summer-born children from autumn, winter and spring-born children (Figure 3A) based on a specific TRF pattern (Figure 3B) with a cross-validated sensitivity and specificity of 0.68 (*p*_permutation,cross-validated_ < 0.001). Specifically, the model identified a higher frequency of TRF 342 bp in summer-born children, along with increased frequencies of TRF 61 bp, TRF 339 bp and TRF 396 bp (Figure 3C) but did not discriminate the richness of nasopharynx species of children born in autumn, winter and spring from each other.

Thus, summer-born children had an overall higher nasopharyngeal bacterial richness represented by, among others, bacterial communities with TRFs at 61 bp, 339 bp, 342 bp and 396 bp. Comparing the discriminatory TRFs with the in silico predicted TRFs received using MiCA [21,22], we identified that TRFs 61 bp, 339 bp and 342 bp could belong to the Gram-negative alpha-proteobacteria class including the order *Rhizobiales*, whereas TRF 396 bp could belong to Gram-positive Firmicutes of the *Bacilli* class. 

## 4. Discussion 

### 4.1. Main Findings

This extensive perinatal exposure analysis revealed that season of birth highly influences nasopharyngeal bacterial richness in 1-month old children. Specifically, summer-born children were found to display an overall higher bacterial richness, involving the alpha-proteobacteria *Rhizobiales* and Firmicutes of the *Bacilli* class (based on in silico predictions of summer-associated TRFs). 

### 4.2. Interpretation

The season of birth’s association with nasopharyngeal bacterial composition and richness in 1-months old asymptomatic neonates may relate to changes in environmental bacterial exposures during different seasons in Northern Europe where Denmark is located, and thus be a phenomenon related to location. According to our surveys, no studies have so far examined the differences in bacterial richness during different seasons at different locations that could justify the summer season-related changes identified in the present study. However, studies examining the outdoor air in urban areas of Italy and the Midwestern United States have found a lower bacterial abundance in winter compared to summer, and a shift in bacterial composition between winter and summer periods [24,25]. Combined with the present data, we speculate that fluctuations in ambient air bacterial communities between seasons may impact the first colonization of the neonatal airways. From the in silico predictions of summer-associated TRFs, it appeared that the Gram-negative alpha-proteobacteria *Rhizobiales* may be present in the nasopharynxes of summer-born children. These plant-associated bacteria are also reported to be common members of the summer atmosphere in northern Italy and the Midwestern United States [24,25]. A remarkably higher prevalence of *Bacillus* and *Lactobacillus* species was also previously identified in the nasopharynxes of 18-months old infants in spring compared to fall/winter [26]. In our study, we likewise found a TRF related to Bacilli classes to be more prevalent in summer children. The nasopharyngeal microbiota diversity has previously been shown to be connected to vitamin D levels, which could be an alternative explanation contributing to the seasonal variability [27].

Season of birth has been proposed as a risk factor for childhood asthma: delivery 4 months prior to the winter virus peak has been associated with increased disease risk [28,29]. We have previously shown that specific pathogenic airway bacteria (*H. influenzae*, *M. catarrhalis* and *S. pneumoniae*) are more prevalent in the hypopharynxes of 1-month old neonates who develop asthma by age 5, and in our newer cohort using 16S rRNA sequencing, we observed similar associations [5], implying that specific airway bacterial exposures early in life can influence the development of asthma. These findings suggest that shaping factors for the 1-month airway colonization are important to study. Furthermore, the airway microbiome in early life has been found very dynamic, changing and diversifying at least until age 3 months [6]. A study has reported increased bacterial diversity in bronchoalveolar lavage from adults with severe asthma [30], thereby suggesting that specific airway bacteria may play a role in chronic asthma as well. 

In agreement with recent studies, gender did not affect nasal bacterial richness [8,31]. Our data further showed that predisposition to asthma and allergies, and specific perinatal exposures, including antibiotic consumption during pregnancy, did not affect the overall nasopharyngeal bacterial richness established at birth. This was in agreement with our previous publication on richness of the gut microbiota in the neonates from the same study cohort [9], where we further showed that increased bacterial diversity in the gut was associated with lower risk of allergic disease. Mode of delivery has also previously been shown to affect the composition of the gut microbiota of the newborn, with less diversity and predominance of skin bacteria being the case in children delivered by caesarean section compared to vaginally-delivered children [32,33,34,35]. However, we found that mode of delivery did not affect the bacterial richness of the neonatal nasopharyngeal region. 

Cumulative data indicate that initial colonizers of the airway mucosa may influence perinatal maturation of the airway epithelium and the immune system, thereby affecting the airway inflammatory status and physiology, and hence resulting in disease risk [3,4,36]. During recent years, several studies have emerged focusing on assessment of the airway microbiota in active airway-related diseases by use of culture-independent techniques. The purpose has been to describe the microbiota dominating in different airway diseases and to depict relationships between commensals and airway pathogens in specific disease settings [8,30,31,37,38,39]. Based on our present study with a large sample size, and the reports by others on 18-month old infants [26], interpretations of bacterial compositions and interspecies correlations to clarify the origins of different airway pathogeneses must be done with great caution unless the studies are well-balanced or adjusted with respect to seasonality of birth and/or sampling time. The current study emphasizes that a future focus on the seasonality aspect in regard to bacterial richness will likely help with modelling the impact of early dynamic changes in airway communities in relation to later disease development. From the literature, it is evident that seasonality is associated with disease risk; however, it is impossible to conclude that a high bacterial richness in an infant’s airways if he/she is summer-born is a good thing. A high richness in airway samples from 1-month old neonates has previously been associated with a higher risk of later asthma [5], but perhaps the seasonal variation in specific bacterial taxa colonizing the airways can exert protection.

### 4.3. Strengths and Limitations of the Study

This study is strengthened by being nested within the COPSAC_2000_ cohort with consistent and accurate longitudinally collected data by trained research staff. Thus, our study is an extensive perinatal exposure assessment in relation to nasopharyngeal bacterial diversity in early life, including a range of factors expected to shape the commensal community, such as furred animals in households [40,41]. 

We observed an overall median bacterial richness of 35 phylogenetically distinct bacterial groups in the nasopharynxes of healthy neonates by using the 16S rRNA gene T-RFLP methodology. This number, as well as the inter-individual variability in richness, is within the range of the operational taxonomic units (distinct individual bacterial sequences) earlier reported in nasopharyngeal samples from healthy infants less than two years of age [26,31]. We recovered 261 different TRFs in the nasopharynx, which aligns with a recent study reporting 179 different TRFs in the oral microbial communities by use of the same T-RFLP-methodology [7]. 

We applied the culture-independent T-RFLP methodology to generate bacterial community profiles and assess bacterial richness within the nasopharynxes of 1-month old, asymptomatic neonates. Although this technique has now been surpassed by next generation sequencing methodologies, data based on the T-RFLP technology are still reliable and can be used to deliver valid information on bacterial richness and diversity [42]. The T-RFLP technique amplifies genes from total community DNA using labelled primers in polymerase chain reaction, followed by digestion with restriction enzymes [43]. Differences in the sizes of resulting TRFs reflect sequence polymorphisms of amplified genes whereby phylogenetically distinct bacterial groups or species are resolved [18]. The T-RFLP technique is constrained by the possibility that multiple bacterial species may be reflected by the same TRF, meaning that the actual nasopharyngeal bacterial richness might be even greater than that identified by means of T-RFLP. This limitation, as well as the fact that experimentally-determined TRF length can differ from in silico determined length [22,44] contribute to uncertainties in the identification of specific bacteria in the microbial community analysis software MiCa [21]. Additionally, the technique has been superseded by other sequencing techniques that allow for precise characterization of the bacterial taxa and quantification. However, our thorough inspection of in silico fragments obtained via MiCa showed that phylogenetically-similar bacteria classes often cluster next to each other, thereby making the approach of in silico prediction of experimentally-derived discriminatory TRFs applicable for initial determination of bacteria genera involved in segregation of data. More elaborate analyses will, however, be required to fully identify the summer-related nasopharyngeal bacteria. 

Our main finding of an association between the airway microbiota and season of birth was robust according to the performed post-validation tests, thereby strengthening the validity of the study’s outcome. The possibility of bias from background noise was reduced by removing TRFs present in <1% of the samples, and, moreover, the seasonal variation persisted even after removal of TRFs present in <10% of the samples. Furthermore, the children within the study cohort were born over two consecutive years, allowing for testing of seasonal consistency in specimen collection, thereby justifying that the birth season-to-diversity association was not biased by sampling in one year only. In addition, by inspection of data we secured that high individual richness in a few samples did not drive the overall bacterial diversity assigned to a season. Previously, the dynamics of the nasal microbiota in infancy were studied in a prospective cohort study of 47 unselected infants [45]. The study revealed seasonality as major factor driving the composition of the nasal microbiota within the first year of life.

The children enrolled in the COPSAC_2000_ cohort were born to mothers with histories of asthma. Although we have no reasons to assume that the birth season-related effect on nasopharyngeal bacterial richness in neonates could be affected by maternal asthma diagnosis, the results would benefit from replication in a population-based cohort, preferentially by applying next generation sequencing [46]. 

Given the observational study design, we cannot evaluate directionality or causality. It remains elusive whether the summer affects the early airway microbiota directly through higher microbial exposures from the environment or through host effects; e.g., a seasonal effect on the infant’s immune system allowing for certain taxa to colonize. 

## 5. Conclusions

This extensive perinatal exposure analysis within the COPSAC_2000_ cohort revealed that season of birth affects the nasopharyngeal bacterial richness of asymptomatic neonates, with summer-born children displaying increased bacterial richness and specific prevalence of bacteria related to Gram-negative alpha-proteobacteria and Gram-positive Bacilli. Increased insight into such decisive factors linking early acquisition of microbial communities to later disease protection will likely enhance the value of future applications and assessments of early intervention strategies to improve human health.

## Figures and Tables

**Table 1:** Associations between perinatal risk factors and TRF richness in the nasopharynxes of asymptomatic neonates.

Variables		No.	No. of Cases (%)	Richness in TRFs Median (IQR)	*p*-Value ^1^
Atopic predisposition					
	Maternal asthma ^2^	328	328 (100)	-	-
	Maternal rhinitis - Yes - No	327	246 (75.2)	36 (15–55) 34 (14–52)	0.67
	Maternal dermatitis - Yes - No	328	163 (49.7)	36 (16–54) 34 (13–56)	0.68
	Paternal asthma - Yes - No	318	51 (16.0)	42 (17–61) 32 (14–55)	0.11
	Paternal rhinitis - Yes - No	316	107 (33.9)	37 (19–53) 32 (12–60)	0.18
	Paternal dermatitis - Yes - No	316	43 (13.6)	45 (22–67) 35 (14–55)	0.044 ^3^
**Social status**					
	Family income, 1000 EUR/year - <53.6 - 53.6–80.4 - >80.4	307	81 (27.4) 149 (48.5) 77 (25.1)	25 (9–49) 40 (18–55) 40 (15–61)	0.031 ^4^
	Mother’s education - College - Elementary/Medium - University	301	179 (59.5) 82 (27.2) 40 (13.3)	35 (16–55) 37 (18–61) 37 (12–55)	0.76
	Mother’s occupation - Unemployed - Non-professional - Professional - Student	306	29 (9.5) 103 (33.7) 143 (46.7) 31 (10.1)	26 (6–45) 31 (16–52) 38 (18–61) 41 (14–58)	0.14
**Pregnancy**					
	Previous deliveries - None - 1 - 2 or more	300	178 (59.3) 91 (30.3) 31 (10.3)	36 (16–55) 41 (15–61) 40 (16–54)	0.91
	Antibiotic use in 3rd trimester - Yes - No	328	44 (13.4)	26 (12–51) 36 (15–55)	0.28
	Paracetamol use in 3rd trimester - Yes - No	328	39 (11.9)	40 (14–67) 33 (14–54)	0.18
	Smoking in 3rd trimester - Yes - No	328	48 (14.6)	27 (17–50) 35 (14–55)	0.59
	Alcohol in 3rd trimester - Yes - No	328	51 (15.5)	25 (14–61) 35 (15–54)	0.86
**Birth**					
	Gender - Female - Male	328	159 (48.5) 169 (51.5)	29 (15–53) 38 (14–60)	0.24
	Mother’s age at birth, years - <25 - 25–35 - ≥35	328	38 (11.6) 238 (72.6) 52 (15.9)	27 (8–47) 36 (15–58) 36 (15–53)	0.14
	Gestational age, weeks - <≤38 - >38 ≤41 - >41	328	66 (20.1) 130 (39.6) 132 (40.2)	29 (13–55) 36 (15–54) 36 (14–57)	0.93
	Mode of delivery - Natural birth - Caesarean section	328	259 (79.0) 69 (21.0)	32 (15–55) 40 (14–55)	0.77
	Apgar score ≥9 at 5min - Yes - No	325	315 (96.9)	35 (14–55) 35 (13–51)	0.95
	Season of birth - Winter - Spring - Summer - Autumn	328	77 (23.5) 69 (21.0) 87 (26.5) 95 (29.0)	25 (14–55) 26 (7–51) 46 (28–69) 31 (12–51)	**0.003 ^5^**
**Postnatal exposures**					
	Cat at birth - Yes - No	303	38 (12.5)	23 (10–61) 37 (15–55)	0.32
	Dog at birth - Yes - No	311	34 (10.9)	34 (20–62) 37 (15–55)	0.46
	Older siblings at birth - None - 1 - 2 or more	309	185 (59.9) 89 (28.8) 35 (11.3)	32 (15–52) 40 (15–61) 40 (17–55)	0.54
	Solely breastfed > 30 days - Yes - No	321	195 (60.7)	37 (15–55) 30 (14–56)	0.67
**Specimen collection**					
	Child age, days - ≤30 - 31–60 -- ≥61	321	35 (10.9) 230 (71.7) 56 (17.4)	17 (9–46) 37 (16–59) 30 (15–53)	0.73

^1^ Statistical analysis by ANOVA for category variables and linear regression for continuous variables. Bonferroni-corrected significance level was 0.002. ^2^ All mothers enrolled in the study had a doctor-diagnosed history of asthma. ^3^ *p*-value = 0.25 after correction for false discovery rate (FDR). *p*-value = 0.15 after adjusting for family income and season of birth. ^4^ *p*-value = 0.24 after correction for FDR. *p*-value = 0.09 after adjusting for father’s dermatitis and season of birth. ^5^ *p*-value = 0.035 after correction for FDR. *p*-value = 0.008 after adjusting for father’s dermatitis and family income.

## Supplementary Materials

The following are available online at https://www.mdpi.com/2227-9067/7/5/45/s1, Figure S1: Bacterial TRF richness variance across different sampling seasons, Table S1: Baseline characteristics of study cohort, Table S2: Bacterial richness of nasopharynx and presence of discriminatory TRFs divided into year of childbirth.

## Governance

We are aware of and comply with recognized codes of good research practice, including the Danish Code of Conduct for Research Integrity. We comply with national and international rules on the safety and rights of patients and healthy subjects, including Good Clinical Practice (GCP) as defined in the EU’s Directive on Good Clinical Practice, the International Conference on Harmonisation’s (ICH) good clinical practice guidelines and the Helsinki Declaration. Privacy is important to us, which is why we follow national and international legislation on General Data Protection Regulation (GDPR), the Danish Act on Processing of Personal Data and the practice of the Danish Data Inspectorate.

## Abbreviations

COPSAC	Copenhagen Prospective Studies on Asthma in Childhood
T-RFLP	terminal restriction fragment length polymorphism
TRFs	terminal restriction fragments
T-REX	T-RFLP analysis *EX*pedited
PLSDA	Partial Least Square Discriminant Analysis
LV	Latent Variable
