# Supplementary material for: Season of Birth Impacts the Neonatal Nasopharyngeal Microbiota
Source: Children (Basel). 2020 May 11;7(5):45. doi: 10.3390/children7050045 (PMC7278723; doi:10.3390/children7050045)
Supplement: Supplementary file 1 [file children-07-00045-s001.pdf]

Supporting Information

Fig S1: Bacterial TRF richness variance across different sampling seasons.

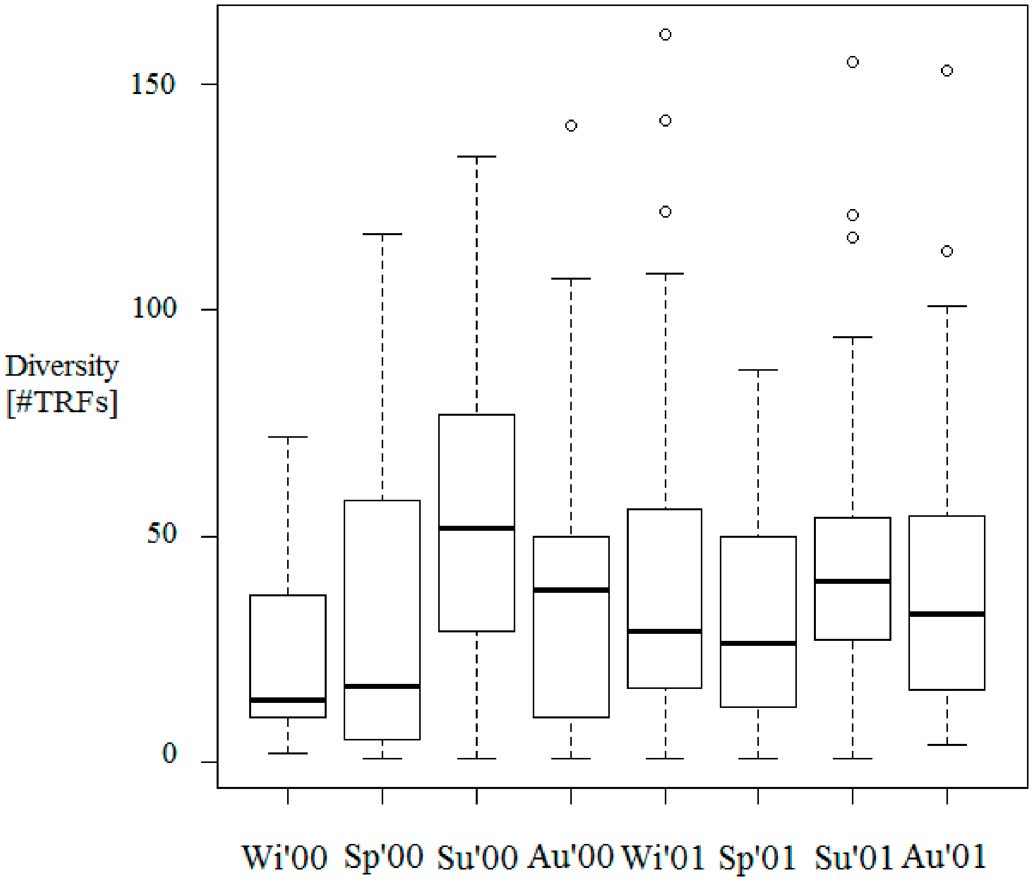

Wi: winter; Sp: spring; Su: summer; Au: autumn; ’00: year 2000; ’01: year 2001

Table S1: Baseline characteristics of study cohort.

		Study cohort	Remaining	P-value
**N**		328	83	
**Heredity**				
	Maternal asthma[1], n (%)	328 (100)	83 (100)	-
	Maternal rhinitis, n (%)	246 (75.0)	64 (77.1)	0.83
	Maternal dermatitis, n (%)	163 (49.7)	35 (42.2)	0.27
	Paternal asthma, n (%)	51 (15.5)	17 (20.5)	0.37
	Paternal rhinitis, n (%)	107 (32.6)	24 (28.9)	0.56
	Paternal dermatitis, n (%)	43 (13.1)	9 (10.8)	0.68
**Social status**				
	Family income[2], n (%)	155 (47.3)	31 (37.3)	0.15
	Mother's highest education[3], n (%)	40 (12.2)	12 (14.5)	0.70
**Pregnancy**				
	Previous deliveries, n (%)	122 (37.2)	29 (34.9)	0.87
	Antibiotic use in 3rd trimester, n (%)	44 (13.4)	15 (18.1)	0.37
	Paracetamol use in 3rd trimester, n (%)	39 (11.8)	20 (24.1)	**0.008**
	Smoking in 3rd trimester, n (%)	48 (14.6)	15 (18.1)	0.54
	Alcohol in 3rd trimester, n (%)	51 (15.5)	22 (26.5)	**0.030**
**Birth**				
	Gender, male, n (%)	169 (51.5)	49 (59.0)	0.11
	Mother's age at birth, years, means (SD)	29.9 (4.4)	30.5 (5.0)	0.30
	Gestational age, weeks, means (SD)	39.9 (1.6)	39.8 (1.6)	0.65
	Head circumference, mm, mean (SD)	352.6 (16.2)	350.5 (16.3)	0.31
	Birth length, cm, mean (SD)	53.5 (2.4)	53.7 (2.3)	0.46
	Birth weight, kg, mean (SD)	4.1 (0.7)	4.2 (0.7)	0.67
	Mode of delivery, Caesarean section, n (%)	69 (21.0)	16 (19.3)	0.95
	APGAR score ≥9 at 5min, n (%)	324 (98.8)	81 (97.6)	1.00
	Season of birth, n (%)			0.97
	- Winter	77 (23.5)	18 (21.7)	
	- Spring	69 (21.0)	17 (20.5)	
	- Summer	87 (26.5)	24 (28.9)	
	- Autumn	95 (29.0)	24 (28.9)	
**Postnatal exposures**				
	Cat at birth, n (%)	38 (11.6)	13 (15.7)	0.26
	Dog at birth, n (%)	34 (10.4)	15 (18.1)	**0.039**
	Older siblings at birth, n (%)	124 (37.8)	28 (33.7)	0.69
	Solely breastfed > 30days, n (%)	195 (59.5)	60 (72.3)	**0.036**

[1]All mothers enrolled in the study had a doctor diagnosed history of asthma
[2]Above 67,000 EUR
[3]Mother's highest education at least university graduate

Table S2: **Bacterial richness of nasopharynx and presence of discriminatory TRFs divided into year of childbirth.**

	**Study cohort**	**Children born in year 2000**	**Children born in year 2001**
N	328	127	182
**Diversity**			
Autumn (median/n)	31/95	38/41	33/39
Spring (median/n)	26/69	17/25	27/44
Summer (median/n)	46/87	52/41	40/46
Winter (median/n)	25/77	14/20	29/53
**Discriminatory TRFs**			
TRF 62 bp	+	+	+
TRF 339 bp	+	+	+
TRF 342 bp	+	+	+
TRF 396 bp	+	-	+
